# Cardiovascular disease risk assessment and multidisciplinary care in prostate cancer treatment with ADT: recommendations from the APMA PCCV expert network

**DOI:** 10.1007/s00345-024-04852-2

**Published:** 2024-03-14

**Authors:** Axel S. Merseburger, Ganesh Bakshi, Dong-Yi Chen, Edmund Chiong, Michel Jabbour, Jae Young Joung, Allen Yu-Hung Lai, Nathan Lawrentschuk, Tuan-Anh Le, Chi Fai Ng, Choon Ta Ng, Teng Aik Ong, Jacob See-Tong Pang, Danny M. Rabah, Narasimhan Ragavan, Kazuhiro Sase, Hiroyoshi Suzuki, Michelle Mui Hian Teo, Hiroji Uemura, Henry H. Woo

**Affiliations:** 1 Department of Urology, University Hospital Schleswig-Holstein, Lübeck, Germany; 2 Department of Surgical Oncology, P. D. Hinduja Hospital and Medical Research Center, Mumbai, India; 3 Division of Cardiology, Department of Internal Medicine, Chang Gung Memorial Hospital, Linkou Branch, Chang Gung University College of Medicine, Taoyuan, Taiwan; 4 Department of Urology, National University Hospital, and Department of Surgery, National University of Singapore, Singapore, Singapore; 5 Division of Urology, Saint Georges Hospital, Balamand University, Achrafieh, Beirut, Lebanon; 6 Center for Urological Cancer, National Cancer Center, Goyang, South Korea; 7 Global Health Program, College of Public Health, National Taiwan University, Taipei, Taiwan; 8 Ferring Pharmaceuticals, Singapore, Singapore; 9 Department of Urology and Department of Surgery, Royal Melbourne Hospital, University of Melbourne, Melbourne, Australia; 10 Cho Ray Hospital, Ho Chi Minh City, Vietnam; 11 SH Ho Urology Centre, Department of Surgery, The Chinese University of Hong Kong, Sha Tin, Hong Kong; 12 Department of Cardiology, National Heart Centre Singapore, Singapore, Singapore; 13 Department of Surgery, Faculty of Medicine, University of Malaya, Kuala Lumpur, Malaysia; 14 Division of Urology, Department of Surgery, Chang Gung Memorial Hospital at Linkou, Taoyuan, Taiwan; 15 The Cancer Research Chair and Department of Surgery, College of Medicine, King Saud University, Riyadh, Saudi Arabia; 16 Department of Urology, King Faisal Specialist Hospital and Research Centre, Riyadh, Saudi Arabia; 17 Department of Urology, The Tamil Nadu Dr MGR Medical University, Apollo Hospitals, Chennai, India; 18 Clinical Pharmacology and Regulatory Science, Graduate School of Medicine, Juntendo University, Tokyo, Japan; 19 Department of Urology, Toho University Sakura Medical Center, Chiba, Japan; 20 Yokohama City University Medical Center, Yokohama, Japan; 21 Department of Urology, Blacktown Hospital, Blacktown, NSW Australia; 22 Department of Uro-Oncology, Chris O.Brien Lifehouse, Camperdown, NSW Australia; 23 College of Health and Medicine, Australian National University, Canberra, ACT Australia

**Keywords:** Cardiovascular disease, Prostate cancer, Interdisciplinary, Risk management, Androgen deprivation therapy, Cardiovascular toxicity

## Abstract

**Purpose:**

Androgen deprivation therapy (ADT) is the mainstay approach for prostate cancer (PCa) management. However, the most commonly used ADT modality, gonadotropin-releasing hormone (GnRH) agonists, has been associated with an increased risk of cardiovascular disease (CVD).

**Methods:**

The PCa Cardiovascular (PCCV) Expert Network, consisting of multinational urologists, cardiologists and oncologists with expertise in managing PCa, convened to discuss challenges to routine cardiovascular risk assessment in PCa management, as well as how to mitigate such risks in the current treatment landscape.

**Results:**

The experts identified several barriers, including lack of awareness, time constraints, challenges in implementing risk assessment tools and difficulties in establishing multidisciplinary teams that include cardiologists. The experts subsequently provided practical recommendations to improve cardio-oncology care for patients with PCa receiving ADT, such as simplifying cardiovascular risk assessment, individualising treatment based on CVD risk categories, establishing multidisciplinary teams and referral networks and fostering active patient engagement. A streamlined cardiovascular risk-stratification tool and a referral/management guide were developed for seamless integration into urologists’ practices and presented herein. The PCCV Expert Network agreed that currently available evidence indicates that GnRH antagonists are associated with a lower risk of CVD than that of GnRH agonists and that GnRH antagonists are preferred for patients with PCa and a high CVD risk.

**Conclusion:**

In summary, this article provides insights and guidance to improve management for patients with PCa undergoing ADT.

## Introduction

Globally, prostate cancer (PCa) is the second-most prevalent cancer and the fifth-leading cause of cancer death amongst men, with an estimated 1.41 million incidence cases and 375,000 deaths. By 2040, these numbers are projected to increase to 2.43 million cases and 740,000 deaths [1, 2]. This burden is compounded by the heightened risk of cardiovascular disease (CVD) amongst patients with PCa [3–5]. The prospective RADICAL PC cohort study revealed that 69% of newly diagnosed patients had a high CVD risk based on the Framingham Risk Score [4], which corresponds to a 10-year CVD event risk of greater than 20% [6]. Indeed, CVD is the leading cause of non-cancer-related death amongst men aged ≥ 40 years with PCa in the United States, accounting for 30.2% of all fatalities [7].

Androgen deprivation therapy (ADT) remains the mainstay approach to PCa management [8, 9], achieved by surgical castration or medical therapy with gonadotropin-releasing hormone (GnRH) agonists, GnRH antagonists, or androgen pathway inhibitors [8]. Although these treatments have improved survival outcomes [10], patients receiving ADT may face a higher CVD risk than that of the PCa-free population [11, 12] and even compared with patients not receiving treatment [13]. A 2010 meta-analysis showed that GnRH agonists led to higher risks of diabetes and CVD than those of men who did not receive GnRH agonists [13]. Manufacturers of GnRH agonists subsequently updated safety labels to communicate these risks [14]. A later study also demonstrated that ≥ 2 years of GnRH agonist use led to a 23% increase in the composite of myocardial infarction and stroke [15].

Interestingly, clinical data have demonstrated that GnRH antagonists are associated with preferable cardiovascular outcomes to GnRH agonists, in addition to having a comparable or potentially superior efficacy profile [16–25]. Randomised controlled trials (RCTs) demonstrated that the incidence of major adverse cardiovascular events (MACE) was lower with GnRH antagonists than with GnRH agonists [16, 17, 22–24]. An analysis of pooled data from six Phase 3 trials (*N* = 2328) showed a 40% relative risk reduction of cardiac events within 1 year of starting GnRH antagonists compared with GnRH agonists, whilst men with pre-existing CVD had a 56% relative risk reduction with GnRH antagonists [16]. Similarly, meta-analyses of eight trials [17], ten trials [22] and 11 trials [25] concluded that GnRH antagonists were associated with lower mortality and cardiovascular events than those of GnRH agonists [17, 22, 25]. Data from the Taiwan National Health Insurance Research Database also found that the risk of MACE and composite cardiovascular events was lower with a GnRH antagonist than with a GnRH agonist amongst patients with pre-existing CVD [18]. Other RCTs [26, 27] and a real-world study [28] have shown the two treatment options to have similar cardiovascular safety profiles.

Further evolution of the treatment landscape has seen novel hormonal agents (NHAs) confer additional oncological benefits when added to ADT [8]. However, NHAs may also be accompanied by an increased CVD risk. Meta-analyses have reported increased cardiac toxicity with abiraterone treatment for metastatic PCa [29, 30], and studies indicate a higher incidence of hypertension and atrial fibrillation with enzalutamide than that with placebo [31, 32]. With future treatment strategies likely to involve a combination of ADT and NHA, a cumulative increase in cardiovascular toxicity is anticipated [33].

Nevertheless, increasing therapeutic options for PCa––with their diverse cardiovascular effects––present an opportunity for personalised treatment [34]. The European Society of Cardiology (ESC) 2022 Guidelines on Cardio-Oncology recommend GnRH antagonists for patients with pre-existing coronary artery disease who require ADT [34]. However, real-world data show that GnRH antagonists are not widely adopted in practice [35, 36], indicating a gap between guidelines and practice that should be bridged by prioritising cardioprotection.

To address this gap, the PCa Cardiovascular (PCCV) Expert Network convened virtually in March 2023 to identify key barriers and develop feasible solutions related to routine cardiovascular risk assessment and mitigation in PCa management. This article presents expert recommendations for assessing and stratifying CVD risk, implementing a multidisciplinary team (MDT) approach and tailoring patient management and surveillance.

### Expert consensus building

The PCCV Expert Network comprised fourteen urologists, three cardiologists and one medical oncologist practising in various countries/regions, including Australia, Germany, Hong Kong, India, Japan, Lebanon, Malaysia, Saudi Arabia, Singapore, South Korea, Taiwan, the United Arab Emirates and Vietnam.

Data on the cardiovascular impact of different PCa treatments were presented during the meeting and served as a foundation for treatment recommendations. Experts also identified barriers to the routine assessment and management of CVD risk, including lack of awareness regarding cardiovascular toxicities associated with ADT; the need for timely initiation of ADT treatment; challenges in using risk assessment tools in busy clinical practice; and difficulties in involving cardiologists in PCa treatment planning. The experts then discussed the feasibility of implementing CVD risk assessment, stratification and management into existing workflows.

Subsequently, the experts proposed recommendations to promote the widespread adoption of CVD risk assessment, treatment options tailored to risk profiles and recommendations for streamlining interdisciplinary referral. It is important to note that the recommendations presented herein are supported by general agreement amongst experts rather than a formal assessment of consensus.

## Discussion and recommendations

### CVD risk assessment

The ESC Guidelines on Cardio-Oncology emphasise the importance of assessing cardiovascular risk associated with cardiotoxic cancer therapies and further recommend the use of the SCORE2 [37] or SCORE2-OP [38] risk assessment tool for cardiovascular risk stratification in patients with PCa [34]. Several cardiovascular risk calculators, including the Framingham Risk Score [6], ESC HeartScore [39], QRISK^®^3 [40], JBS3 risk calculator [41], and the ACC/AHA Atherosclerotic Cardiovascular Disease (ASCVD) Risk Estimator [42], may also be used by urologists as alternative tools for assessing CVD risk.

**Fig. 1:**
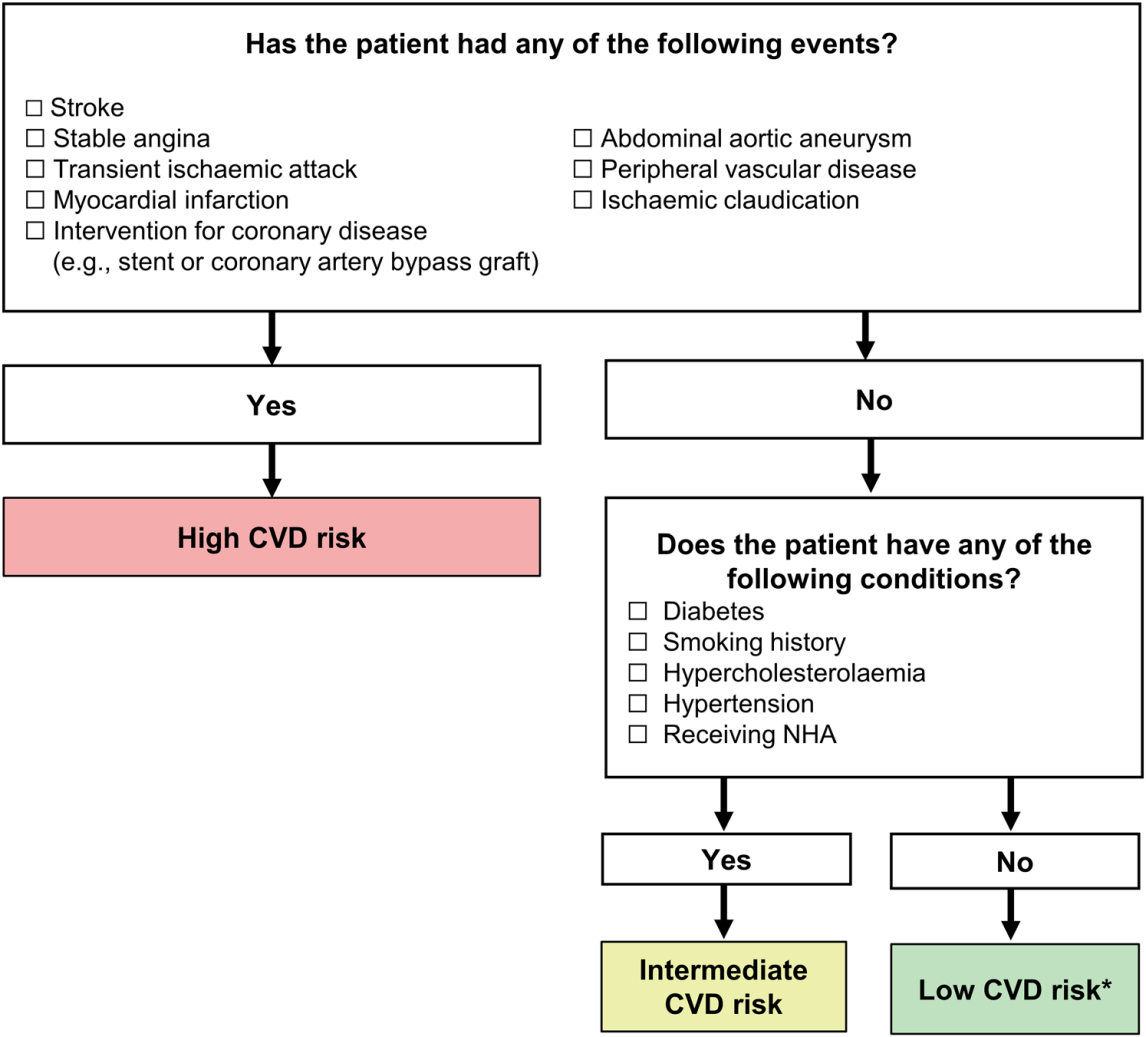
Checklist for CVD risk assessment and stratification. Adapted from Davey P and Alexandrou K. Int J Clin Pract; 2022 [3]. A minimum of one check-marked condition is needed to select “Yes” in a subsequent text box. *A patient’s risk level may transition from “Low Risk” to “Intermediate Risk” or “High Risk” after 2 or 3 years of hormonal plus NHA treatment. CVD, cardiovascular disease; NHA, novel hormonal agent; PCa, prostate cancer

The PCCV panel indicated that the routine adoption of these existing cardiovascular risk calculators is hindered by the perception that these tools are cumbersome and may not be practical for busy urologists. Instead, urologists mainly rely on subjective cardiovascular health assessment (e.g. eye-balling) during in-clinic physical examination, which involves evaluation of medical history and symptoms. The PCCV panel proposed integrating a simplified and objective cardiovascular risk assessment tool into treatment decision workflows of urologists. Figure 1 presents a combined checklist and risk stratification tool, which has been adapted from an algorithm developed by Davey and Alexandrou [3]. This tool provides a practical framework that allows urologists to promptly estimate patients’ CVD risks based on common cardiovascular risk factors and stratifies patients into the following three risk categories: low, intermediate, or high. It is recommended that urologists conduct CVD risk assessment and stratification before initiating ADT treatment. However, patients with ongoing ADT treatment may also benefit from CVD risk assessment, because elevated risk may warrant medication review.

The PCCV panel emphasised that the CVD risk categories depicted in Fig. 1 are solely intended to guide subsequent management and should not be misconstrued as a predictor of future cardiovascular events, unlike the Framingham Risk Score [6]. To enhance its utility, the checklist shown in Fig. 1 can be transformed into a printed leaflet that patients can complete in the waiting room, with assistance from clinic nurses, healthcare staff or the patients’ caregivers. Such checklists should be presented in layman terms as well as local languages, to facilitate patient comprehension [43].

**Table:** 

*PCCV Expert Network recommendation*: A simplified checklist can be seamlessly integrated into clinical practice and facilitate the objective assessment of CVD risk by urologists.	

### Impact of CVD risk on decision-making

**Fig. 2:**
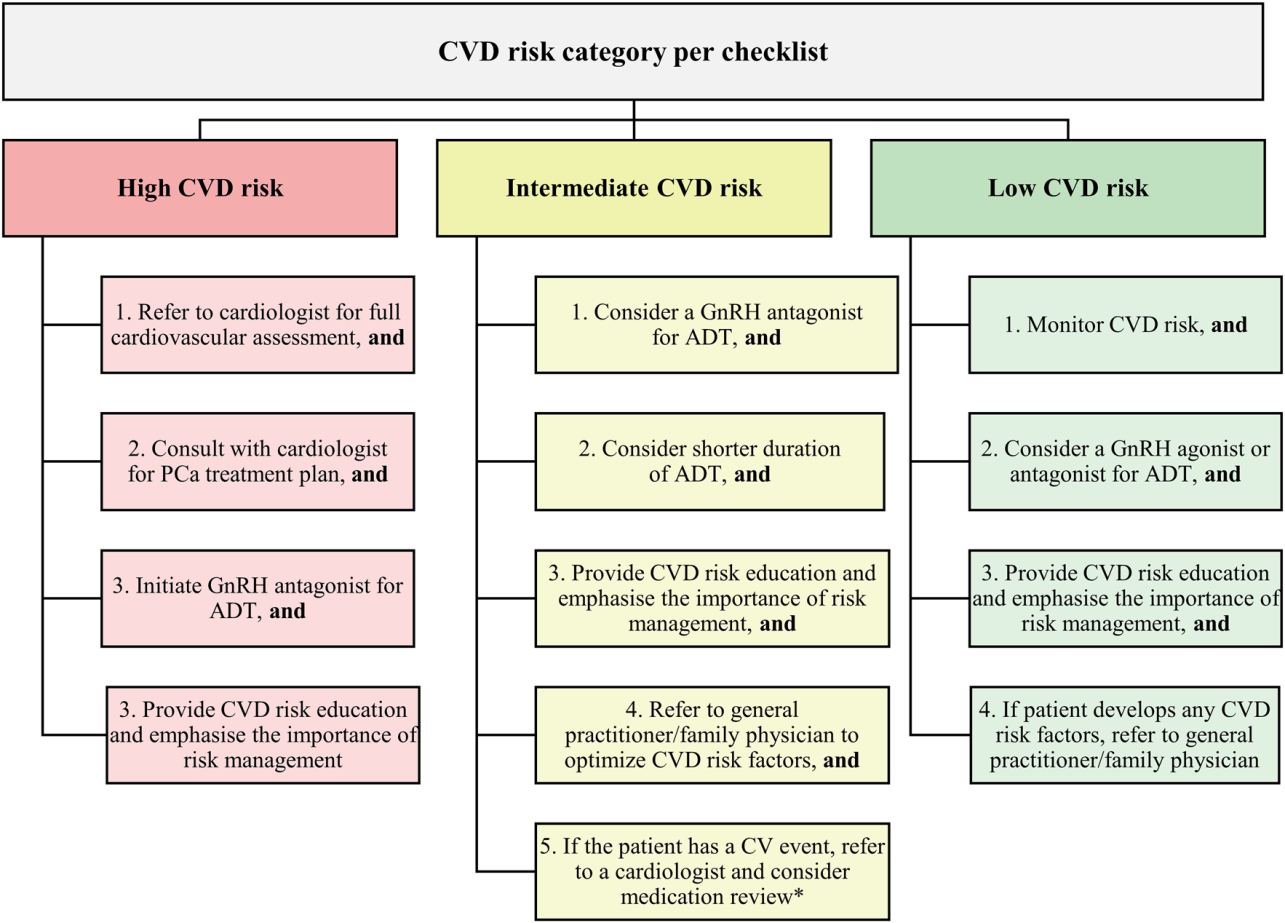
Management steps for minimising cumulative CVD risk at ADT initiation. *Referral to cardiologists is recommended but is subject to each country’s healthcare system and resources. ADT, androgen deprivation therapy; CV, cardiovascular; CVD, cardiovascular disease; GnRH, gonadotrophin-releasing hormone; PCa, prostate cancer

The ESC Guidelines recommend that physicians tailor PCa treatment to the cardiovascular health of patients and consider CV toxicities of individual ADTs [34]. Figure 2, adapted from an algorithm developed by Davey and Alexandrou [3], illustrates PCCV panel recommendations for immediate next steps based on the CVD risk category determined in Fig. 1.

Studies indicate that men in developing countries often present with a more advanced stage of PCa [44, 45], necessitating prompt intervention. Therefore, for patients with low CVD risk, ADT should be initiated as soon as possible. Patients with intermediate or high CVD risk should ideally be subject to efforts to optimise risk factors before, during and after cancer treatment. For patients with active cardiac symptoms, cardiologist referral can be considered before treatment initiation to optimise cardiovascular health management and minimise treatment disruption/discontinuation owing to future cardiovascular events. For patients with pre-existing CVDs, having the highest risk of future cardiovascular events [46], the PCCV panel agreed that GnRH antagonists should be administered in accordance with guideline recommendations [34].

It is important to carefully consider the most suitable ADT class for patients who may benefit from a combination treatment of NHA and ADT, with the aim to minimise the cumulative CVD risk. To our knowledge, there is a scarcity of RCTs comparing the cardiovascular burden of GnRH agonists and antagonists when each is used in combination with an NHA. However, as evidence suggests that GnRH antagonists are associated with lesser risk of CV toxicities than those of GnRH agonists [16–24], GnRH antagonists should be considered to minimise cumulative cardiovascular risk of combination treatment.

**Table:** 

*PCCV Expert Network recommendation*: GnRH antagonists are the preferred ADT for patients with high CVD risk.	

### Multidisciplinary care

An MDT-based approach is ideal for PCa management; that is, in addition to the treating urologist, care may also be provided by cardiologists, medical oncologists, radiation oncologists, general practitioners and family physicians [47–49].

The PCCV panel highlighted that cardiologists are currently underrepresented in MDTs for several reasons. First, urologists may be unaware of the importance of cardiologist consultation before ADT initiation in patients at high CVD risk. Second, urologists may lack access to on-site cardiologists or are unacquainted with cardiologists who specialise in cardiotoxicity. Lastly, urologists may assume that cardiologists lack the time or interest to participate in management decisions. However, considering the substantial CVD burden in patients with PCa, there is a clear need for a shared-care approach [34].

**Fig. 3:**
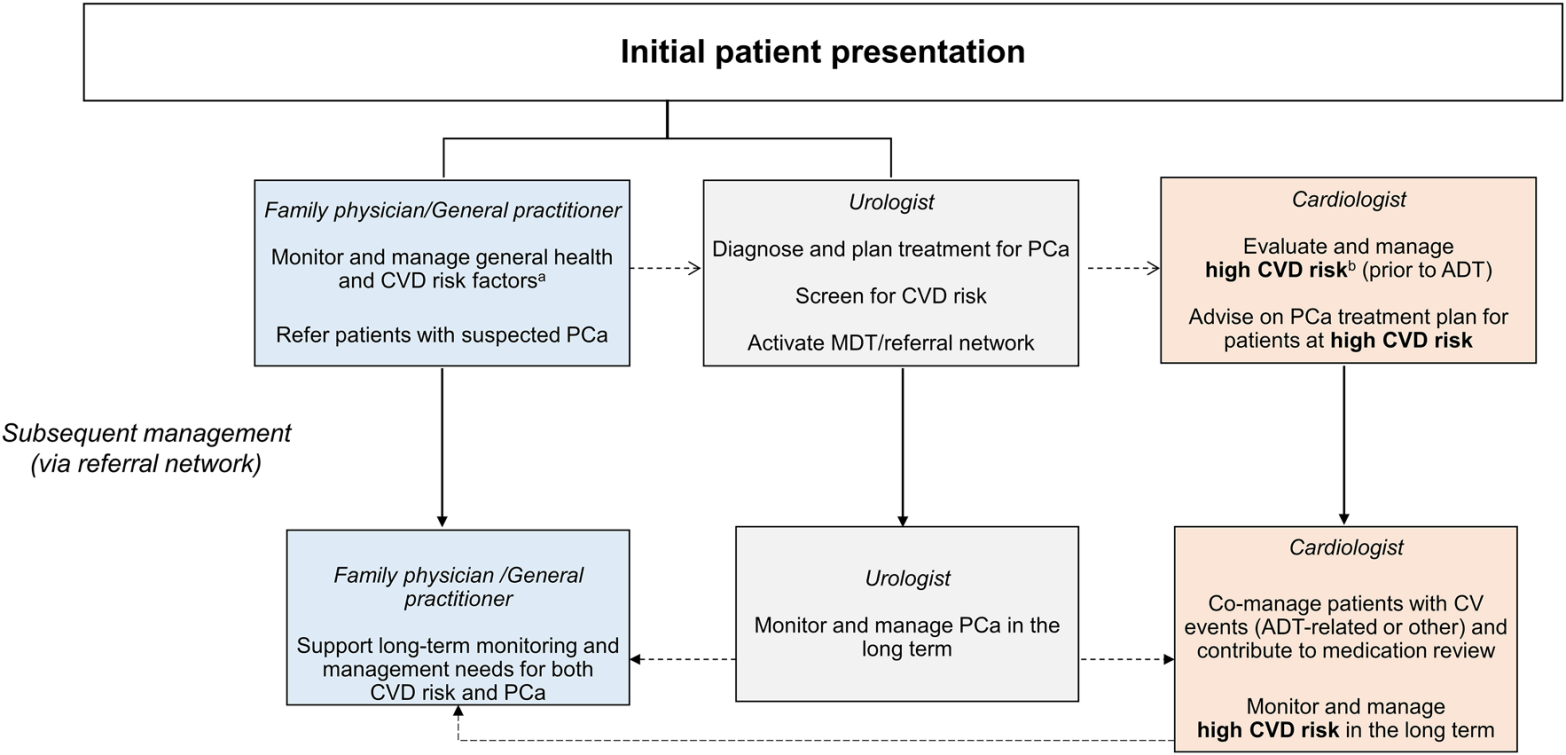
Optimal workflow for the multidisciplinary management of PCa and CVD. Dotted arrows indicate the necessity of interdisciplinary co-management of patients, highlighting the importance of ongoing communication between healthcare disciplines throughout the management of PCa. There is a need for interdisciplinary communication for the duration of management of PCa, especially in cases where high CVD risk is observed at diagnosis. ^a^Include treatment of diabetes or hyperlipidaemia, smoking cessation, regular exercise, weight reduction to BMI < 25 kg/m^2^. ^b^Include heart attack in the past 1 year, ongoing chest pain or discomfort. ADT, androgen deprivation therapy; BMI, body mass index; CV, cardiovascular; CVD, cardiovascular disease; MDT, multidisciplinary team; PCa, prostate cancer.

The PCCV panel has proposed a workflow for optimising PCa management (Fig. 3), taking into consideration the potential cardiovascular effects of ADT and the importance of cardiologist assessment, particularly for patients at high CVD risk. To minimise the risk of treatment disruption/discontinuation owing to cardiovascular events, urologists are advised to pre-identify cardiologists with expertise or interest in cardio-oncology, for timely patient referrals and input into treatment plans.

Other pivotal HCPs are general practitioners and family physicians, who often refer patients to urologists in the first instance, communicating the patients’ medical and treatment history and conducting initial cardiovascular risk assessments. General practitioners and family physicians also provide ongoing treatment of comorbid conditions, including diabetes, hypercholesterolaemia, smoking and hypertension.

In some countries, patients may consult with a urologist directly, without a prior referral. If a urologist identifies such patients as having high CVD risk, the urologist should ideally refer the patient to a cardiologist for further evaluation before initiating ADT treatment [3]. Conversely, if the urologist determines that a patient is at lower CVD risk, immediate PCa treatment should be considered. In addition, these patients should be referred to a general practitioner or a family physician (or as necessary, a cardiologist) for comprehensive assessment, including measurements of blood pressure, lipids, fasting glucose, glycated haemoglobin and an electrocardiogram, and for comorbidity management. Moreover, patients should receive guidance for controlling CVD risk factors.

Whilst Fig. 3 highlights the potential roles of physicians alone, the contribution of uro-oncology nurses should be acknowledged. Uro-oncology nurses offer patient insights from a holistic perspective, including psychosocial considerations, that may inform and complement medical treatment strategies [48, 50]. Indeed, MDTs that involve nursing staff appear more effective than teams without nurse representation [48].

**Table:** 

*PCCV Expert Network recommendation*: An MDT-based approach should be implemented, to optimise cardiovascular assessment, treatment planning and patient care.	

### Communication within the MDT

When patients are responsible for the identification of specialists and coordination of appointments for their cancer care, the communications between HCPs are typically uncoordinated and fragmented [51]. In addition, the need for urgent cardiovascular assessment may not be adequately communicated by the patient.

Urologists are encouraged to proactively establish referral networks and build strong relationships with other specialists. Cardio-oncology societies can be a valuable platform for building referral networks and establishing rapport between specialists. Although establishing this network may require considerable upfront effort, it offers access to specialised guidance and diverse medical attention [52].

Urologists are also advised to determine an effective means of communication with other specialists, such as telemedicine or other digital platforms, for systematic information sharing, timely review of documentation and routine follow-up. Asynchronous communication is beneficial when scheduling conflicts, geographical constraints, or time limitations impede direct or immediate communication between specialists. However, wherever feasible, asynchronous communications should be supplemented with face-to-face discussion, which provides more timely resolution of disagreements in treatment planning.

**Table:** 

*PCCV Expert Network recommendation*: Urologists are encouraged to establish interdisciplinary referral networks for timely and specialised care.	

### Long-term MDT management of PCa

Following treatment initiation, the urologist should develop a comprehensive plan for long-term care, which should involve monitoring and managing both PCa and any comorbid conditions, as well as ensuring treatment adherence. Proactive monitoring and management of cardiometabolic adverse events of PCa treatment should also be taken into account. For example, for patients who are at risk of QTc prolongation with ADT, baseline and serial electrocardiogram assessments are recommended [34]. Anticipating intolerable or severe side effects, the urologist should pre-identify alternative treatment options.

Workflows for long-term PCa management that tap into the expertise of multiple specialities not only provide the patient with comprehensive care, but also ease the burden on urologists, so they may focus on providing optimal and uninterrupted oncological treatment. For example, general practitioners or family physicians may be responsible for monitoring adherence and adverse events, supporting ongoing cancer surveillance and managing comorbid conditions. Practical and financial considerations provide further rationale for patients to interact more frequently with general practitioners or family physicians than urologists over the treatment course. However, these primary care providers commonly report communication gaps and loss of patient contact after referral; often, they are not routinely copied on patient reports from specialists [53]. Communication challenges may be overcome through the use of digital platforms that facilitate ongoing interdisciplinary collaboration.

**Table:** 

*PCCV Expert Network recommendation*: Urologists should proactively communicate with general practitioners or family physicians for awareness of cardiovascular events that emerge in parallel with, or owing to, PCa treatment.	

### Long-term MDT management of CVD risk

The PCCV panel indicated that urologists currently face an undue burden of responsibility for monitoring both cardiovascular health and PCa. As described earlier, an optimal workflow should involve strategically allocating responsibilities amongst the referral network.

For patients classified as having low-to-intermediate CVD risk during screening, general practitioners or family physicians may be assigned the responsibility of annual cardiovascular assessment and monitoring patient adherence with the cardiovascular health plan. A comprehensive multidisciplinary ABCDE approach, which includes risk assessment, blood pressure control, cholesterol management, diabetes care and tailored exercise prescription, can be utilised as a structured means to optimise cardiovascular well-being [49]. If a patient experiences a cardiovascular event or exhibits abnormalities during cardiovascular assessment, the general practitioner or family physician should inform the urologist, as it may necessitate a medication review and referral to a cardiologist.

Patients with high CVD risk may require close, specialised cardiovascular monitoring and care, to be determined by a cardiologist and communicated to the healthcare team (Fig. 3). Relevant specialists should devise a new surveillance plan encompassing both cancer recurrence and cardiovascular health [49]. Cardiovascular surveillance and vigilance should be maintained throughout the ADT course.

**Table:** 

*PCCV Expert Network recommendation*: Cardiovascular events typically warrant a referral to a cardiologist and adjustment of PCa treatment plans.	

### Maintaining patient engagement

Clinicians are well aware of the challenges for achieving patient adherence to management plans for chronic diseases [54, 55]. Evidence indicates that patients who are educated about their condition and actively involved in their management plan achieve better disease control than those who lack information and opportunities for engagement with HCPs [56, 57]. A qualitative interview study underscoring the diverse requirements of patients with PCa concluded that HCPs should instil patients with a sense of empowerment and provide support mechanisms to facilitate the decision-making process [58]. For example, providing a booklet that includes cardiovascular health education and facilitates self-recording of vital signs, test results and lifestyle goals throughout the treatment journey, which can increase patient accountability, as well as facilitate communication of health data with other treating physicians [57].

Sustained support from HCPs, in the form of regular encouragement, acknowledgement of achieved goals and personalised advice, has also been identified as a means for achieving adherence [55, 57]. This approach may allow patients to maintain their commitment to their cardiovascular health and potentially improve health outcomes.

**Table:** 

*PCCV Expert Network recommendation*: Patient engagement strategies can foster long-term patient adherence to the management plan.	

### Limitations of the review

There are currently limited insights regarding the specific impact of ADT choice, such as GnRH agonists versus GnRH antagonists, on overall CVD risk for patients who have well-controlled cardiovascular risk factors at baseline. We acknowledge the current evidence gap and thus refrain from recommending a specific ADT to be initiated for patients with low CVD risk during assessment. We also acknowledge the inherent limitations associated with the ‘expert consensus’ methodology, such as potential bias in the selection of experts, who may have greater resources for implementing cardiovascular management plans in routine practice than the community urologists in the region. However, it should be noted that the PCCV panel comprises specialists from diverse cultural backgrounds and practice settings to capture comprehensive insights into PCa management in the Asia–Pacific region.

Another limitation of this study is that a formal assessment of expert agreement was not utilised, such as the Delphi method or a predetermined ‘cut-off’ to indicate agreement with each recommendation. Nevertheless, clinical strategies which received vocal disagreement during the meeting were not recommended in this article, such as routine implementation of formal CVD risk calculators. Whilst we recognise that incorporating quantitative metrics could offer a more concrete measure of agreement, we feel that the qualitative approach that was adopted could provide insightful expert recommendations for consideration by the wider medical community.

### Future directions

The field of cardio-oncology is gaining prominence owing to an increasing awareness of the potential cardiotoxicity of cancer treatments and an aging population, accompanied by a higher prevalence of comorbidities [59]. To the best of our knowledge, there are currently limited RCTs evaluating the clinical outcomes of tailoring ADT selection to CVD risk categories, particularly for patients with a low-to-intermediate CVD risk. Further research is needed to assess the management strategies proposed in this article, and real-world evidence will likely be how such strategies can be explored. Medical education emphasising the feasibility of CVD risk assessment and mitigation in routine PCa care may enhance the adoption of these practices. In addition, educational meetings such as an MDT tumour board could be valuable platforms for fostering collaboration amongst professionals across specialities, with the aim of facilitating in-person networking and establishing local referral networks.

Other educational campaigns could be directed at expanding the role of general practitioners or family physicians in ongoing cancer care, such as training in subcutaneous administration of GnRH antagonists. This can reduce the frequency of hospital visits, thus alleviating the burden on patients and urologists, whilst providing opportunities for regular monitoring of cardiovascular health.

The multidisciplinary approach described herein aims to enhance coordination amongst HCPs of different specialities. Research is needed to evaluate the feasibility and pharmacoeconomic impact of collaborative cardiovascular health management in PCa care, which could help justify its adoption in national health policies and reimbursement schemes.

## Conclusion

There is a growing demand to evolve current PCa treatment strategies to account for comorbidities, particularly pre-existing CVD or risk factors. Raising awareness of cardiovascular risk factors and implementing routine risk assessment during consultations are essential components of long-term management of PCa. Simple tools have been devised to support risk stratification and decision-making; however, further research is required to compare the cardiovascular risks of various PCa treatments. Meanwhile, proactive collaboration and communication between health-care providers can drive positive change in the field of PCa treatment and ultimately improve patient outcomes.

## Data Availability

No new data were created or analyzed in this study. Data sharing does not apply to this article.
